# Physiotherapy Treatment in Patients with Hemophilia and Chronic Ankle Arthropathy: A Systematic Review

**DOI:** 10.1155/2013/305249

**Published:** 2013-08-12

**Authors:** Rubén Cuesta-Barriuso, Antonia Gómez-Conesa, José Antonio López-Pina

**Affiliations:** ^1^Research Group in Physiotherapy and Health Promotion, Regional Campus of International Excellence “Campus Mare Nostrum”, University of Murcia, 30100 Murcia, Spain; ^2^Department of Basic Psychology and Methodology, Faculty of Medicine, Espinardo Campus, University of Murcia, 30100 Murcia, Spain

## Abstract

Haemophilic arthropathy of the ankle causes pain and deterioration in gait, causing disability. Although some physiotherapy modalities are effective in the management of acute bleeding, the results are unknown in chronic arthropathy. Our objective was to determine the most effective physiotherapy procedures for treating the haemophilic arthropathy of the ankle and to assess the methodological quality of the studies. A systematic review was carried out in the Cochrane Database, PubMed, MEDLINE, ISI Web of Knowledge, PEDro, TESEO, and specialized journals (Haemophilia and Haematologica). It included articles with at least one group undergoing any kind of physiotherapy treatment and with pretest and posttest evaluation, published before April 2013. An analysis of variables was performed and assessed the methodological quality of studies. Five studies met the criteria for inclusion. Hydrotherapy treatments, strength training and balance strength, balance training, and sports therapy, have improved range of movement, pain, balance, and subjective physical performance. The proposed methodological analysis was not possible due to the low quality of the studies. Although the results are positive, they lack rigorous evidence on the effects of treatments. Studies are needed to establish the efficacy of the various forms of physiotherapy in the haemophilic arthropathy of the ankle.

## 1. Introduction

Hemophilia is a blood-clotting disorder caused by a deficiency in factor VIII (FVIII) or factor IX (FIX), which manifests itself through bleeding in the muscles and joints [1]. There are three categories, depending on the percentage of the blood-clotting factor: severe hemophilia (<1% FVIII/IX), characterised by spontaneous bleeding; moderate hemophilia (1–5% FVIII/FIX), with bleeding from slight injuries; and mild hemophilia (>5–40% FVIII/FIX) with bleeding during surgical procedures or from severe injuries [2, 3].

Eighty percent of the episodes in hemophiliac patients involve bleeding in the joints, or haemarthrosis [4, 5], with the ankle being the third most frequently affected joint [6]. The symptoms of intra-articular ankle bleeding are severe pain, limited range of motion, inflammation, and synovial involvement. Without proper treatment, capsular and tendon contractures can develop in the joints [7].

Although experiments on animals cannot be directly related to the humans, Hooiveld et al. [8] observed in a canine model that weight-bearing joints, as opposed to non-weight-bearing joints with haemarthrosis, suffered progressive and degenerative damage after a bleeding episode. Also analysing the effects of haemarthrosis, Hakobyan et al. [9] tested in a murine model the effect of the ferric component of blood, in vitro, on the development of chronic synovitis. 

Excess blood inside the joint results in inflammation of the synovial membrane, which eventually leads to chronic haemophilic synovitis and a vicious cycle of haemarthrosis-synovitis-haemarthrosis [15].

When synovitis becomes chronic, the condition is worsened by the recurrent haemarthrosis episodes, accelerating the degenerative process known as haemophilic arthropathy, which leads to alterations of the joints, pain, muscular atrophy, and functional impairment [16]. Chronic arthropathy is a major cause of morbidity in patients with haemophilia [17].

Due to the progression of haemophilic arthropathy and the resulting deterioration of the joint, deformities such as the restriction of motion, valgus hindfoot, alteration of the subtalar and tibiofibular-astragalar joints, or plano-valgus foot [18] occur. Likewise, the chronic pain associated with haemophilic arthropathy is a good predictor of disability in patients with severe haemophilia [19].

Current pharmacological treatments prophylactic with FVIII or FIX significantly reduce the frequency of haemarthrosis in hemophilia. This decrease in the incidence of joint and muscle bleeding, spontaneous and posttraumatic, has delayed the incidence of haemophilic arthropathy [20, 21]. When arthropathy is already instituted, this treatment can only slow the joint deterioration but not prevent it [22, 23].

On the other hand, the 80% of hemophilia patients have no access to pharmacological therapy [24], and part of the remaining 20% only receive treatment after a bleeding episode (on demand treatment). In both cases, patients develop physical consequences before reaching adulthood, that require physiotherapy treatment to improve and maintain joint function [25].

Physiotherapy through the RICE method (Rest, Ice, Compression, and Elevation) has been described for the improvement of acute joint injuries [26], as well as in the management of hemarthrosis in patients with hemophilia [27].

Treatment of the ankle with physical therapy is complicated because it involves a small joint surface that bears significant ranges of body weight and the joint limitation alters biomechanical movement during walking. A treatment combining strength and proprioception exercises improved functionality in a group of 31 patients with haemophilic arthropathy on knee and ankle [10].

Likewise, for the ankle joint, the guide of the World Federation of Haemophilia recommends exercises to range of movement, strength, and balance [28], while these recommendations are not based on any clinical study.

Even though it is a joint with a high prevalence of degenerative lesions [29], it is not known how significant or efficacious physical therapy is in managing arthropathy of the ankle.

The main objective of this work is to learn from the existing literature about the effectiveness of physical therapy procedures in treating chronic arthropathy of the ankle in patients with hemophilia. It will also analyse the methodological quality of the studies and propose new lines of research.

## 2. Methods

### 2.1. Data Sources and Searches

The literature search was performed according to the search strategy described by Dickersin et al. [30].

Combined processes were developed for the study search, establishing the following steps: (I) the following databases of specialised literature were consulted: Cochrane, MEDLINE, PubMed, PEDro, TESEO and ISI Web of Knowledge; (II) the search ended in May of 2013; (III) the medical subjects heading included “hemophilia” AND “ankle” AND “arthropathy” AND “rehabilitation” OR “physiotherapy” OR “physical therapy” OR “TENS” OR “transcutaneous” OR “hydrotherapy” OR “kinesiotherapy” OR “manual therapy” in the article, and specialised electronic magazines were consulted: HaemophiliaandHaematologica.

We also reviewed bibliographies of relevant papers, conference messages, dissertations, and consultations with experts. Two authors reviewed the abstracts and full texts of the publications found in the databases and journals, and if in doubt, the eligibility of any of the articles was determined by consensus.

### 2.2. Study Selection

The studies selected met the following criteria: (I) they use physical therapy treatments and (II) clinical trials; (III) they include at least one treatment group with pretest and posttest evaluations; (IV) the size of the sample in the posttest is a minimum of five individuals per group; (V) years considered: without restrictions in the year of publication; the study had to have been published prior to April 2013; (VI) the studies included are restricted to those in Spanish, French, English, Italian, and Portuguese, (VII) studies without limit of age, and (VIII) articles published in scientific journals or in process of publication; (IX) case studies are excluded, (X) as are those in which the individuals were not diagnosed with hemophilia A or B, or (XI) those in which the physical therapy treatment used was not explained. 

**Figure 1:**
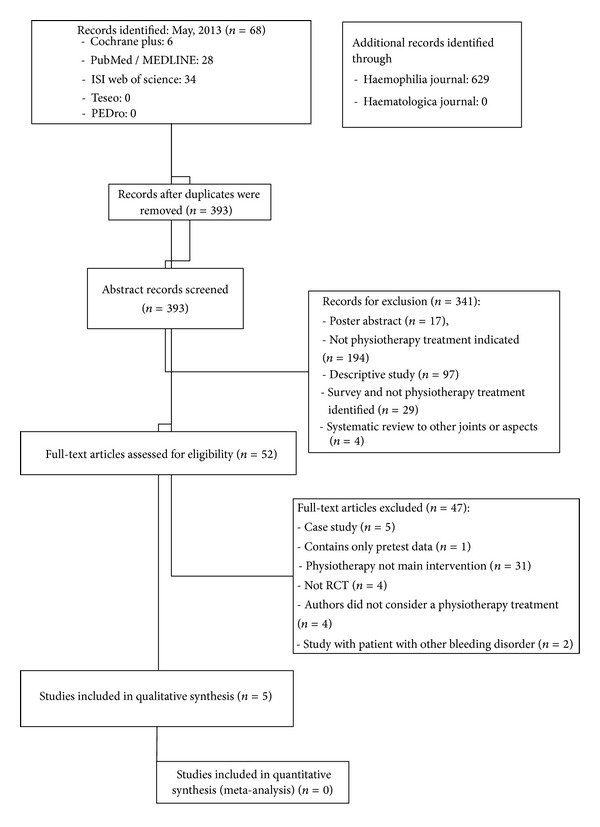


After the search, in which more than 600 references were consulted, a total of five articles [10–14] fulfilled the inclusion criteria.  Figure 1 shows the results of the search and selection process.

### 2.3. Encoding of the Variables

It was performed according to the procedure for coding the studies of the Meta-Analysis Unit of Murcia University (http://www.um.es/metaanalysis/presentation.php/).

The characteristics of the five selected studies were encoded. The moderating variables were grouped in three categories in accordance with the Lipsey et al. [31] classification system: substantive variables (treatment, context, and individual involved), methodological variables, and extrinsic variables.

The encoded characteristics of the treatment were the following: the type of physical therapy treatment (muscular strength, proprioception training, hydrotherapy, and sports therapy); the duration of treatment (in weeks); the intensity of the treatment (number of hours for session); the extent of the treatment (total number of hours per patient); the number of sessions; inclusion of the follow-up programme (in weeks); the uniformity of the treatments (patients from the same group receive the same treatment); the mode of intervention: direct (applied by a physiotherapist), indirect (under the supervision of a physiotherapist), or mixed; and informed consent.

The encoded characteristics of the individuals were the following: the average age (in years) of the individuals in the sample; the type of hemophilia; the severity of the hemophilia; and whether or not prophylactic drug treatment with FVIII/FIX was used. The encoded characteristics of the context were country and place of intervention (hospital, university, etc.). The extrinsic characteristics that were encoded were: (a) the date of the study (year) and (b) the source of publication (published versus not published).

### 2.4. Methodological Quality and Measuring the Results

To evaluate the methodological quality of the studies, we used the Van Tulder et al. [32] and PEDro [33] scales, which are designed to evaluate randomized clinical trials with a view to conducting systematic reviews.

To evaluate the impact of the different physical therapy procedures used to treat arthropathy of the ankle, the effect size of the studies was reviewed using the Rosenthal formula [34]. 

## 3. Results

### 3.1. Study Selection

The initial search of electronic databases provided 68 articles. Through the additional search of specialized electronic journals and consultation with experts, 629 articles were located. We reviewed 393 abstracts of which 52 went to full-text review. Finally, 5 studies met all inclusion criteria.

### 3.2. Participants

Finally, they were 94 patients with hemophilia and 47 people without congenital coagulopathies. All patients were males, diagnosed with hemophilia A or B, with different phenotypes of gravity, based on the percentage of FVIII/FIX in blood.

### 3.3. Characteristics of the Studies

We analysed the five studies that met the inclusion criteria.  Table 1 contains the most relevant results of the studies analysed, and the characteristics of the potentially moderating variables are presented in Tables 2 and 3.

Regarding the treatment variables, we found different types of intervention, distinguishing between a combination of muscle strength training and proprioception [10, 11, 13], hydrotherapy [12] and sports therapy [14].

With respect to the quantitative treatment variables, the intervention lasted an average of 20 weeks; each individual received an average of 10 hours of treatment per week, and each individual received treatment totalling an average of 120 hours.

Regarding the variables for the individuals, just three of the studies [10, 13, 14] indicated the type and severity of the hemophilia in the sample. The Hill et al. [13] study examined 19 patients with hemophilia A and one with hemophilia B, of whom 14 had the severe, four had the moderate, and one had the mild phenotype. In the Gurcay et al. [10] sample, on the other hand, there were 25 patients with hemophilia A and six with hemophilia B, of whom 21 had the moderate, six had the severe, and four had the mild phenotype. Czepa et al. [14] examined 24 patients with hemophilia A and one with hemophilia B, of whom 21 had the severe and 3 had the moderate phenotype. The prophylactic treatment was covered by the authors in two articles [10, 13]. In the Czepa et al. [14] study, patients were on pharmacological regimen provided for their haematologist (12/25).

Regarding the extrinsic characteristics, all of the studies are from journal articles. One study was conducted in 2003 [11] and the other four in 2008, 2009, 2010, and 2013 [10, 12–14].

### 3.4. Methodological Quality and Measuring the Results

Of the five studies, two had no control group [10, 13], and in the other two, the control group consisted of individuals who were either healthy [11] or with no ankle pathology [12]. These studies have no control group with the same characteristics that the treatment group (patients with hemophilia and ankle arthropathy), and none of the scales anticipated in the design of this review could be applied (Van Tulder and PEDro).

In the study of Czepa et al. [14], the control group (passive PWH) had the same characteristics that the active group. Tables 4 and 5 show assessment of these articles with Van Tulder and PEDro scales.

The size effect could not be calculated as anticipated because of the lack of a control group or because of the latter's characteristics.

### 3.5. Measuring Instruments

The studies selected for this review used a wide variety of clinimetric measures to detect a measurable change after the physiotherapy treatment. Gurcay et al. [10] evaluated ROM (with a universal goniometer); perception of pain (scale of joint pain, score 0–3): functional incapacity (Juvenile Arthritis Functional Assessment Report for Children; JAFAR-C questionnaire); and the clinical evaluation (evaluation scale adopted by the Orthopaedic Advisory Committee of the World Federation of Haemophilia).

The Hilberg et al. [11] study evaluated proprioception (test: One-Leg Stand, angle reproduction, tuning fork, and Posturomed) and isometric muscular strength (test: knee extensor, and leg press). Garcia et al. [12] evaluated ROM (with a universal goniometer). Finally, the Hill et al. [13] study evaluated proprioception (Functional Reach test, step test, timed up and go, modified Clinical Test of Sensory Interaction on Balance-mCTSIB test and Limits of stability-LOS test); walking (test: Walking across the long plate and Step and quick turn); physical activity (Human Activity Profile questionnaire); fear of falling (modified Falls Efficacy Scale—MFES questionnaire); and the perception of pain (Visual Analogue Scale, or VAS). Czepa et al. [14] evaluated activity level (scale from 0-not active to 4-active more than three times/week); ROM of knee, ankle, and elbow (with a universal goniometer); One-Leg Stand test; and 12 min walk test.

Pretest and posttest evaluations were performed in the five studies [10–14], and Garcia et al. [12] measured ROM before and after each session.

### 3.6. Adverse Effects

Gurcay et al. [10] showed that during treatment with strength and proprioception, there were two patients with hemophilia who developed joint bleeds, while treatments of Hilberg et al. [11], Hill et al. [13], and Czepa et al. [14] did not cause haemarthrosis during the study period, and Garcia et al. [12] provided no data on this respect. 

## 4. Discussion 

This review explored the changes in objective measures of physical therapy treatments in patients with hemophilia and chronic arthropathy of the ankle. The five studies were selected because they provided pretest/posttest data in a treatment group. The studies reviewed examined the impact of different physical therapy treatments on the clinical manifestations of chronic arthropathy of the ankle (reduced ROM, loss of strength, pain, and alteration of proprioception) in patients with hemophilia. Three studies were conducted in a hospital setting [10–12] and two in hospital and in the home [13, 14].

The studies are very heterogeneous with respect to the duration, intensity, and extent of the treatments. The treatments lasted for four weeks [10, 12], sixteen weeks [13], twenty-four weeks [11], and fifty-two weeks [14]; and the number of hours per week for each patient was one hour [12], four hours [11], or five hours [10].

The treatments administered were strength training and, proprioception [10, 11, 13], hydrotherapy [12], and sports therapy [14].

Regarding the occurrence of joint bleeding as a result of physical therapy treatments, in three trials there was no haemarthrosis of the ankle [11, 13, 14] which indicates the safety of the strengthening treatments and sports therapy with exercises adapted to the patients' health status in preventing recurrent bleeding.

These results agree with those of Koch et al. [35] that after treatment with strength training in young people with hemophilia from 12 to 14 years, improvement was noted in quadriceps muscle strength with a decrease in the frequency of haemarthrosis in the knee joint and in the results of Pelletier et al. [36] in a patient with hemophilic arthropathy of the knee treated with isometric exercises. Other authors have also noted that physical activity can improve muscle strength and flexibility and reduce the number of bleeding, synovitis and joint destruction in patients with hemophilia [37].

Development of arthropathy affects the proprioception of patients with haemophilia [38]. After the treatments described in the studies, proprioception improves as a result of strength and proprioception training programmes for the lower limbs [11, 13].

In the ROM evaluation, described by Harris and Boggio [39] as a measure of joint deterioration in patients with hemophilia, significant improvement was found in the evaluation of the ankle after treatment with strength and proprioception exercises [10] and hydrotherapy [12].

Perception of pain is one of the main causes of incapacity in patients with hemophilia [24]; there was significant improvement after strength and proprioception treatment [10, 13].

The treatments of the Gurcay et al. [10] and Hill et al. [13] studies seem to have more effectiveness, with improvement in a larger number of clinimetric measures, although the Gurcay et al. [10] study achieves good results in less time: four weeks as opposed to the 16 required by Hill et al. [13]. However, due to the lack of follow-up periods in the studies of this review, it was not possible to learn whether the effect of the treatments holds up over time.

In the study of Czepa et al. [14], 47.9% of patients dropout the study due to irregular training frequencies, operations during intervention period, private reasons, or patient who entered later into the study. In the study by Hill et al. [13], 40% of patients discontinued due to lack of time, other diseases that affect their ability to participate, lack of motivation or interest in the program, or not believing in the benefits of treatment. The dropout rate is similar to that described in the study by Mulvany et al. [40] who developed physiotherapy treatments for improving muscle strength and flexibility, and cardiovascular function in 33 patients with congenital coagulopathies (hemophilia and von Willebrand disease), and had a 39% dropout due to problems with transportation and schedules and the disease, although no patient reported any adverse effects as a result of treatment. By contrast, there was a lower experimental mortality rate (17.85%) in the study by Hilberg et al. [11], and no patient stopped treatment in the other two studies [10, 12].

### 4.1. Methodological Quality and Measuring the Results

With respect to methodological quality and measuring the results, the analysis of the methodological quality using the Van Tulder and PEDro scales and of effect size, is precluded by the fact there is no control group in the studies with the same characteristics as the experimental group (hemophilia and ankle arthropathy). Only it was possible to assess the methodological quality of the article by Czepa et al. [14] with 2 and 3 points in Van Tulder and PEDro scales, respectively.

Among the articles on physiotherapy treatment of haemophilic arthropathy of the ankle excluded from this review because they did not meet the selection criteria, there are two unique cases [22, 41] one that did not involve posttreatment evaluation [42] and two articles that evaluated the isolated placement of a plantar orthoses [43] and performance of sports without supervision of physiotherapist [39]. Although acupuncture has been used as a treatment of physiotherapy, the article of Lambing et al. [44] has not been included in this review because the authors do not apply it as a physiotherapy treatment. Two articles [40, 45], despite their good methodological quality, include patients with other coagulation deficits (von Willebrand disease-vWD).

Similarly, despite the good methodological quality of the study of Von Mackensen et al's [46], this article was not included in this review because the authors did not assess any physical variable in ankles with hemophilic arthropathy.

### 4.2. Limits of the Review

There were a number of limits placed on this review. The lack of precision in the studies in describing the physical therapy methods and techniques used, as well as the sample data, raised doubts for us during the encoding process. Moreover, the lack of data on moderating variables (randomisation of the sample, masking, and the treatment application method) limited the possibilities for obtaining detailed results on this review.

The low methodological quality of the studies did not permit a detailed analysis of them, nor could outcome measures be extracted to calculate the effect size.

### 4.3. Implications for the Clinical Practice

Results of this systematic review confirm the need for treatment of physiotherapy in the multidisciplinary approach to patients with hemophilia, raised previously by other authors [24, 36]. However, a clear indication about the most efficacious type of treatment is limited by the lack of controlled studies.

Physical therapy treatments must combine various aspects. On the one hand, there must be efficacious treatment of the problems caused by arthropathy of the ankle [42], such as restrictions on range of motion, loss of muscular strength, pain, and alteration of proprioception. On the other hand, the treatment must not cause haemarthrosis. 

### 4.4. Implication for Future Studies

Properly designed randomised clinical studies must be developed for physical therapy treatment in patients with hemophilic arthropathy of the ankle. Furthermore, the study samples must be as broad as possible, and the outcome measures must be valid and reliable. 

Studies with a follow-up period are necessary to establish the efficacy of the treatment in the medium term.

Future studies must describe the characteristics of the individuals with respect to age, weight, the joints affected, the degree of joint impairment, and so forth; the treatments characteristics (procedures, duration, intensity, etc.); and the potential adverse effects in the locomotor system as a result of the treatment.

## 5. Conclusion

There is little uniformity in the studies reviewed with respect to duration, intensity, extent, and types of treatment.

Treatments with strength and proprioception training, hydrotherapy, and sports therapy improve some clinical aspects in patients with hemophilic arthropathy of the ankle before and after comparison.

There is no rigorous evidence on the effects of the treatments.

Methodological quality of the studies is low.

## Figures and Tables

**Table 1:** Details of the studies included in the review (min: minutes).

	Gurcay et al. (2008) [10]	Hilberg et al. (2003) [11]	Garcia et al. (2009) [12]	Hill et al. (2010) [13]	Czepa et al. (2013) [14]
Type of study	Pretest-posttest study	Pretest-postest study with control group being not homogeneus	Pretest-postest study with control group being not homogeneus	Pretest-postest study	Pretest-postest study with two control groups

Participants	31 patients with hemophilia. Mean age: 13.02 y	9 patients with hemophilia (experimental group), 8 subjects without hemophilia (active control group, AC), and 11 subjects without hemophilia (passive control group, PC). Mean age: 34.33 y	9 patients with hemophilia and arthropathy (experimental group) and 9 patients with no pathology or articular anomaly. Mean age: 9 y	20 patients with hemophilia. Mean age: 39.4 y	13 patients with hemophilia (active PwH), 12 patients with hemophilia (passive PwH), and 19 subjects without hemophilia (controls)

Experimental versus control intervention (no. of participants)	Isometric, isotonic, strengthening, and proprioception exercise (*n* = 31)	Strength and propioception training (*n* = 9); strength and propioception training—active control group—(*n* = 8); versus care as usual (*n* = 11)	Vertical bicycle, ability to control breathing, ability to control the rotations, and activities for balance, buoyancy, and immobility control (*n* = 9); same intervention in control group (*n* = 9)	Balance and lower limb strength exercises and a walking programme (*n* = 12)	Sports therapy with exercise to increase focal points in terms of body awareness, muscle tone regulation, joint mobilization, and muscle activation

Duration	4 weeks	24 weeks	4 weeks	16 weeks	52 weeks

Frequency	5 times/week	2 times/week	2 times/week	5–7 times/week	1 time/week (0.98 point in scale 0–4)

Intensity	300 minutes/week	240 minutes/week	60 minutes/week	No data	No data

Outcomes of experimental group	Significant changes in flexion and dorsiflexion (*P* = 0.005) and inversion and eversion (*P* = 0.007) of ankle. Improvement in pain, disability, and clinical assessment (*P* < 0.001).	One-leg-stand test: differences between H and PC groups (*P* < 0.05) and H and AC groups (*P* < 0.05). Angle-reproduction test: differences between H and PC groups (*P* < 0.05). Tuning fork test: differences between H and PS groups (*P* < 0.05) and H and AC groups (*P* < 0.05)	Significant changes in the ROM of left ankle in hemophilia ankle: between sessions (*P* = 0.015) and between all sessions (*P* = 0.011).	Significant changes in static balance (*P* < 0.025); dynamic bilateral stance balance (*P* < 0.017); dynamic single limb balance (*P* < 0.05); gait and mobility (*P* < 0.01); activity (*P* < 0.05); and fear of falling (*P* < 0.05).	Active PwH reported significant changes in subjective physical performance in the HEP-test-Q domains “strength and coordination,” “endurance,” and “body perception” as well as the total score compared to the other groups. A longer walking distance for the active PwH compared with controls in the 12 min walk test.

Measuring instruments	Goniometer (ROM), Jafar-C (functional disability), EVA (pain. score 0–3), and clinical evaluation score (adopted by WFH)	One-leg-stand test, Posturomed test, angle reproduction test, and tuning fork test (propioception); strength test (isometric muscular strength)	Goniometer (ROM)	Functional Reach test, step test, and timed up and go test (clinical measures of balance); mCTSIB test and LOS test (laboratory measures of balance); walking across the long plate test and step and quick turn (gait measures); Human Activity Profile (physical activity); MFES (falls Efficacy); and VAS (pain. score 0–10)	Activity level (scale 0–4), goniometer (ROM), one-leg-stand test, and 12 min walk test

Pharmacology Cointervention	Prophylaxis treatment (2-3 times/week)	Prophylaxis treatment (if necessary)	No data	No data	Patients were on prophylaxis or on demand treatment, according to medical criteria

Adverse effects	2 patients developed hemarthrosis during treatment	No patient developed hemarthrosis during treatment	No data	No patient developed hemarthrosis during treatment.	No patient developed haemarthrosis during treatment.

**Table 2:** Description of the qualitative moderating variables.

Variables	*k*		Frequency	%
Treatment variables				
Type of treatment	5	ST + PT	3	60
		H	1	20
		SPORT	1	20
Mode of application	5	Direct	2	40
		Indirect	2	40
		Mixed	1	20
Informed consent	5	Yes	3	50
		No	2	50

Context variables				
Country	5	Germany	2	25
		Brazil	1	25
		Australia	1	25
		Turkey	1	25
Place	5	Hospital	3	75
		Domiciliary	2	25

Methodological variables				
Pretest	5	Yes	5	100
		No	0	0
Assignment	5	Random	0	0
		Not random	5	100
Control group	5	Yes	1	0
		No	4	100
Date	5	2003	1	25
		2005–2013	4	75
Published	5	Yes	5	100
		No	0	0
Source	5	Journal article	5	100

*k*: number of studies; ST: strength training; PT: proprioception training; H: hydrotherapy; SPORT: sports therapy.

**Table 3:** Description of the quantitative moderating variables: duration (weeks); intensity (hours/week); magnitude (total hours); SS (size of the sample).

Moderating variables	*k*	Min.	Max.	Average	Median	DT
Treatment variables						
Duration	5	4	52	20	16	19.79
Intensity	3	1	5	3.33	4.00	2.081
Magnitude	3	4	96	40.00	20.00	49.153
Subject variables						
Age (years)	4	13.02	45	32.45	35.90	13.944
Methodological variables						
SS treatment group pretest	5	9	35	18.80	20	10.73
SS treatment group posttest	5	9	31	14.80	12	9.23
SS treatment group followup	0					
SS control group pretest	1	0	27	5.40	0	12.074
SS control group postest	1	0	12	2.40	0	5.366
SS control group followup	0					
Postest differential mortality (%)	5	0	52.80	20.8444	11.42	24.17
Differential mortality followup (%)	0					

**Table 4:** Analysis of the methodological quality of Czepa et al.'s [14] study, by Van Tulder scale.

Items	Czepa et al. (2013) [14]
Was the method of randomization adequate?	No
Was the treatment allocation concealed?	No
Were the groups similar at baseline regarding the most important prognostic indicators?	Yes
Was the patient blinded to the intervention?	No
Was the care provider blinded to the intervention?	No
Was the outcome assessor blinded to the intervention?	No
Were cointerventions avoided or similar?	No
Was the compliance acceptable in all groups?	No
Was the drop-out rate described and acceptable?	No
Was the timing of the outcome assessment in all groups similar?	Yes
Did the analysis include an intention-to-treat analysis?	No

**Table 5:** Analysis of the methodological quality of Czepa et al.'s [14] study, by PEDro scale.

Items	Czepa et al. (2013) [14]
Eligibility criteria were specified	No
Subjects were randomly allocated to groups (in a crossover study, subjects were randomly allocated an order in which treatments were received)	No
Allocation was concealed	No
The groups were similar at baseline regarding the most important prognostic indicators	Yes
There was blinding of all subjects	No
There was blinding of all therapists who administered the therapy	No
There was blinding of all assessors who measured at least one key outcome	No
Measures of at least one key outcome were obtained from more than 85% of the subjects initially allocated to groups	No
All subjects for whom outcome measures were available received the treatment or control condition as allocated or, where this was not the case, data for at least one key outcome was analysed by “intention to treat”	No
The results of between-group statistical comparisons are reported for at least one key outcome	Yes
The study provides both point measures and measures of variability for at least one key outcome	Yes

## References

[1] Molho P, Rolland N, Lebrun T (2000). Epidemiological survey of the orthopaedic status of severe haemophilia A and B patients in France. *Haemophilia*.

[2] Montgomery RR, Scott JP, Nathan DG, Oski FA (1992). Hemostasis: diseases of the Xuid phase. *Hematology of Infancy and Childhood*.

[3] Upchurch KS, Brettler DB, Ruddy S, Harris ED, Sledge CB (2001). Hemophilic arthropathy. *Kelley's Textbook of Rheumatology*.

[4] Duthie RB, Matthews J, Rizza C (1972). *The Management of Musculoskeletal Problems in the Haemophilias*.

[5] Rodriguez-Merchan EC (1996). Effects of hemophilia on articulations of children and adults. *Clinical Orthopaedics and Related Research*.

[6] Hoskinson J, Duthie RB (1978). Management of musculoskeletal problems in the hemophilias. *Orthopedic Clinics of North America*.

[7] Ribbans WJ, Rees JL (1999). Management of equinus contractures of the ankle in haemophilia. *Haemophilia*.

[8] Hooiveld MJJ, Roosendaal G, Jacobs KMG (2004). Initiation of degenerative joint damage by experimental bleeding combined with loading of the joint: a possible mechanism of hemophilic arthropathy. *Arthritis and Rheumatism*.

[9] Hakobyan N, Kazarian T, Jabbar AA, Jabbar KJ, Valentino LA (2004). Pathobiology of hemophilic synovitis I: overexpression of mdm2 oncogene. *Blood*.

[10] Gurcay E, Eksioglu E, Ezer U, Cakir B, Cakci A (2008). A prospective series of musculoskeletal system rehabilitation of arthropathic joints in young male hemophilic patients. *Rheumatology International*.

[11] Hilberg T, Hersbsleb M, Puta C, Gabriel HHW, Schramm W (2003). Physical training increases isometric muscular strength and proprioceptive performance in haemophilic subjects. *Haemophilia*.

[12] Garcia MK, Capusso A, Montans D, Massad E, Battistella LR (2009). Variations of the articular mobility of elbows, knees and ankles in patients with severe haemophilia submitted to free active movimentation in a pool with warm water. *Haemophilia*.

[13] Hill K, Fearn M, Williams S (2010). Effectiveness of a balance training home exercise programme for adults with haemophilia: a pilot study. *Haemophilia*.

[14] Czepa D, von Mackensen S, Hilberg T (2013). Haemophilia & Exercise Project (HEP): the impact of 1-year sports therapy programme on physical performance in adult haemophilia patients. *Haemophilia*.

[15] Rodriguez-Merchan EC (2006). The haemophilic ankle. *Haemophilia*.

[16] Stephensen D, Tait R, Brodie N (2009). Changing patterns of bleeding in patients with severe haemophilia A. *Haemophilia*.

[17] Funk M, Schmidt H, Escuriola-Ettingshausen C (1998). Radiological and orthopedic score in pediatric hemophilic patients with early and late prophylaxis. *Annals of Hematology*.

[18] Pearce MS, Smith MA, Savidge GF (1994). Supramalleolar trial osteotomy for haemophilic arthropathy of the ankle. *Journal of Bone and Joint Surgery Series B*.

[19] van Genderen FR, Fischer K, Heijnen L (2006). Pain and functional limitations in patients with severe haemophilia. *Haemophilia*.

[20] Fischer K, Van Der Bom JG, Molho P (2002). Prophylactic versus on-demand treatment strategies for severe haemophilia: a comparison of costs and long-term outcome. *Haemophilia*.

[21] Royal S, Schramm W, Berntorp E (2002). Quality-of-life differences between prophylactic and on-demand factor replacement therapy in European haemophilia patients. *Haemophilia*.

[22] Tiktinsky R, Falk B, Heim M, Martinovitz U (2002). The effect of resistance training on the frequency of bleeding in haemophilia patients: a pilot study. *Haemophilia*.

[23] Aledort LM, Haschmeyer RH, Pettersson H (1994). A longitudinal study of orthopaedic outcomes for severe factor-VIII-deficient haemophiliacs. *Journal of Internal Medicine*.

[24] Buzzard BM (1999). Physiotherapy for the prevention of articular contraction in haemophilia. *Haemophilia*.

[25] Aznar JA, Magallón M, Querol F, Gorina E, Tusell JM (2000). The orthopaedic status of severe haemophiliacs in Spain. *Haemophilia*.

[26] Bleakley CM, O’Connor S, Tully MA, Rocke LG, MacAuley DC, McDonough SM (2007). The PRICE study (Protection Rest Ice Compression Elevation): design of a randomised controlled trial comparing standard versus cryokinetic ice applications in the management of acute ankle sprain [ISRCTN13903946]. *BMC Musculoskeletal Disorders*.

[27] Hermans C, De Moerloose P, Fischer K (2011). Management of acute haemarthrosis in haemophilia A without inhibitors: literature review, European survey and recommendations. *Haemophilia*.

[28] Mulder K (2006). *Exercise for People with Hemophilia*.

[29] Gamble JG, Bellah J, Rinsky LA, Glader B (1991). Arthropathy of the ankle in hemophilia. *Journal of Bone and Joint Surgery Series A*.

[30] Dickersin K, Scherer R, Lefebvre C (1994). Identifying relevant studies for systematic reviews. *British Medical Journal*.

[31] Lipsey MW, Cooper HM, Hedges LV, Valentine JC (2009). Identifying interesting variables and analysis opportunities. *The Handbook of Research Synthesis and Meta-Analysis*.

[32] Van Tulder M, Furlan A, Bombardier C, Bouter L (2003). Updated method guidelines for systematic reviews in the Cochrane Collaboration Back Review Group. *Spine*.

[33] Maher CG, Sherrington C, Herbert RD, Moseley AM, Elkins M (2003). Reliability of the PEDro scale for rating quality of randomized controlled trials. *Physical Therapy*.

[34] Rosenthal R (1984). *Meta-Analytic Procedures for Social Research*.

[35] Koch B, Cohen S, Luban NC, Eng G (1982). Hemophiliac knee: rehabilitation techniques. *Archives of Physical Medicine and Rehabilitation*.

[36] Pelletier JR, Findley TW, Gemma SA (1987). Isometric exercise for an individual with hemophilic arthropathy. *Physical Therapy*.

[37] Nazzaro A-M, Owens S, Hoots WK, Larson KL (2006). Knowledge, attitudes, and behaviors of youths in the US hemophilia population: results of a national survey. *American Journal of Public Health*.

[38] Hilberg T, Herbsleb M, Gabriel HHW, Jeschke D, Schramm W (2001). Proprioception and isometric muscular strength in haemophilic subjects. *Haemophilia*.

[39] Harris S, Boggio LN (2006). Exercise may decrease further destruction in the adult haemophilic joint. *Haemophilia*.

[40] Mulvany R, Zucker-Levin AR, Jeng M (2010). Effects of a 6-Week, individualized, supervised exercise program for people with bleeding disorders and hemophilic arthritis. *Physical Therapy*.

[41] Luterek M, Baranowski M, Zakiewicz W, Biel A, Pedzisz P (2009). PNF-based rehabilitation in patients with severe haemophilic arthropathy—case study. *Ortopedia Traumatologia Rehabilitacja*.

[42] Heijnen L, de Kleijn P (1999). Physiotherapy for the treatment of articular contractures in haemophilia. *Haemophilia*.

[43] Filho DJ, Battistella LR, Lourenço C (2006). Computerized pedobarography in the characterization of ankle-foot instabilities of haemophilic patients. *Haemophilia*.

[44] Lambing A, Kohn-Converse B, Hanagavadi S, Varma V (2012). Use of acupuncture in the management of chronic haemophilia pain. *Haemophilia*.

[45] d’Young IA (2008). Domiciliary application of CryoCuff in severe haemophilia: qualitative questionnaire and clinical audit. *Haemophilia*.

[46] Von Mackensen S, Eifrig B, Zäch D, Kalnins J, Wieloch A, Zeller W (2012). The impact of a specific aqua-training for adult haemophilic patients—results of the WATERCISE study (WAT-QoL). *Haemophilia*.

